# From Fish Eggs to Fish Name: Caviar Species Discrimination by COIBar-RFLP, an Efficient Molecular Approach to Detect Fraud in the Caviar Trade

**DOI:** 10.3390/molecules24132468

**Published:** 2019-07-05

**Authors:** Anna Maria Pappalardo, Agnese Petraccioli, Teresa Capriglione, Venera Ferrito

**Affiliations:** 1 Department of Biological, Geological and Environmental Sciences–Section of Animal Biology “M. La Greca”, University of Catania, Via Androne 81, 95124 Catania, Italy; 2 Department of Biology, University of Naples “Federico II”, Via Cinthia Ed7, 80126 Naples, Italy

**Keywords:** caviar, species identification, DNA barcoding, COIBar-RFLP

## Abstract

The demand for caviar is growing as is its price on the market. Due to the decline of true caviar production from sturgeons, eggs from other fish species and other animals have been used as substitutes for caviar. The labels on these products should indicate the species from which the eggs were derived, but the label can be misleading in some cases. In this context, species identification using DNA analysis is crucial for traceability and authentication of caviar products. In this work, we applied the COIBar-RFLP procedure to obtain species-specific endonuclease restriction patterns useful to discriminate “caviar” species. The tested caviar products were identified as originating from eight species: *Acipenser transmontanus*, *A. gueldenstaedtii*, *A. stellatus*, *A. baerii*, *Mallotus villosus*, *Huso huso*, *Cyclopterus lumpus* and *Eumicrotremus orbis*. The results demonstrated that 14% of the caviar products examined have a label that does not indicate the species from which the eggs were originated. The *Mbo*I restriction enzyme produced specific profiles discriminating the eight species, confirming that the COIBar-RFLP is a useful approach for routine screening of seafood products due to its ease and rapid execution, as the results of screening can be obtained within 7 h, by-passing the need for sequencing.

## 1. Introduction

Sturgeon eggs, known as “caviar”, represent one of the most valuable delicacies on the world’s food market. As of 1997, all sturgeon species were included in the Appendices of the Convention on International Trade in Endangered Species (CITES). Since then, sturgeon species identification has been a challenge of primary importance [1]. The standard for sturgeon caviar [2], adopted in 2010, indicates that caviar is the product made from fish eggs, treated with grade salt, of the Acipenseridae family (including the genera *Acipenser*, *Huso*, *Pseudoscaphirhynchus* and *Scaphirhynchus*). In regards to the labeling of commercial products, the term “caviar” must be used only for sturgeon eggs and labels should include the common name of the species, such as Beluga for *Huso huso*, Osetra for *Acipenser gueldenstaedtii/A. persicus* and Sevruga for *Acipenser transmontanus*. Furthermore, caviar products include not only the aforementioned true caviar, but also caviar substitutes obtained from eggs of other fish species or other animal organisms, and a variety of other products called “caviar” that have no trace of fish eggs but simulate the taste of caviar [3]. Of course, the prices of the various types of caviar vary considerably, with only true caviar considered a luxury high-priced product (sold for about 900–1500 euro per kilo depending on the sturgeon species, but beluga caviar has also been sold for as much as 4500 euro per kilo). Caviar substitutes are sold at more affordable prices (generally less than 200 euro per kilo). The limited production of true caviar due to the drastic reduction in native sturgeon populations and the global growing demand of caviar products, has led to an increase in both the aquaculture production of sturgeon and caviar on the one hand, and the use of caviar substitutes on the other. In fact, the latter include the eggs of at least 38 non-sturgeon fish species [3]. In this context, there is a high risk of fraud, poaching and illegal trade of caviar, and while the amount of illegal trade is unknown, it has been estimated by the CITES Secretariat that levels of poaching far exceed legal harvesting. The European countries are particularly interested in this problem due to the fact that the EU is the world’s largest importer of caviar from the exporters states such as China, which accounted for 85% of EU imports in 2016 [4].

Currently, a growing scientific literature on seafood product authentication has demonstrated that the highly automated biomolecular techniques can greatly improve species identification in processed seafood products. This is especially relevant because of industrial processing, which leads to the loss of identifiable morphological characters of the species [5,6,7]. Together with advances in large-scale integrated molecular technology, many assays based on DNA analysis have been developed for species identification in several food products. Among them, DNA barcoding, targeting a fragment of about 650 bp of the mitochondrial cytochrome oxidase I (COI) gene, is currently being used to differentiate and discriminate more than 98% of animal species [8,9,10,11,12,13,14,15,16,17,18,19] and has been validated for forensic species identification [20]. However, most of the DNA-based techniques adopted for the authentication of fish species have focused on a high sensitivity and short analysis time to make species identification quick and easy for routine screening for detection of seafood product mislabeling [21,22,23,24].

PCR-RFLP, for example, is a simple, inexpensive, reliable and promising technique, as polymorphic markers are generated by PCR amplification followed by restriction digestion with endonucleases [25,26,27,28]. Recently, the two consolidated approaches of COI barcodes and PCR-RFLP were combined in a new molecular strategy (COIBar-RFLP, cytochrome oxidase I barcode-restriction fragment length polymorphism) for fish species identification in processed seafood products [29,30,31,32]. PCR-RFLP of the cytochrome b gene has already been used to discriminate caviar species, but exhibited limitations in the differentiation of closely related species, such as *A. gueldenstaedti*, *A. baerii*, *A. persicus* and *A. naccarii* [33]. Based on the considerations above, to assess the efficacy of the COIBar-RFLP in caviar species identification, we first applied conventional DNA barcoding to verify the labeling accuracy of several “caviar” products and then performed the COIBar-RFLP molecular strategy to reduce both analysis costs and time of fish species authentication for the most common commercial caviar products.

## 2. Results

### 2.1. COI Sequences

**Figure 1:**
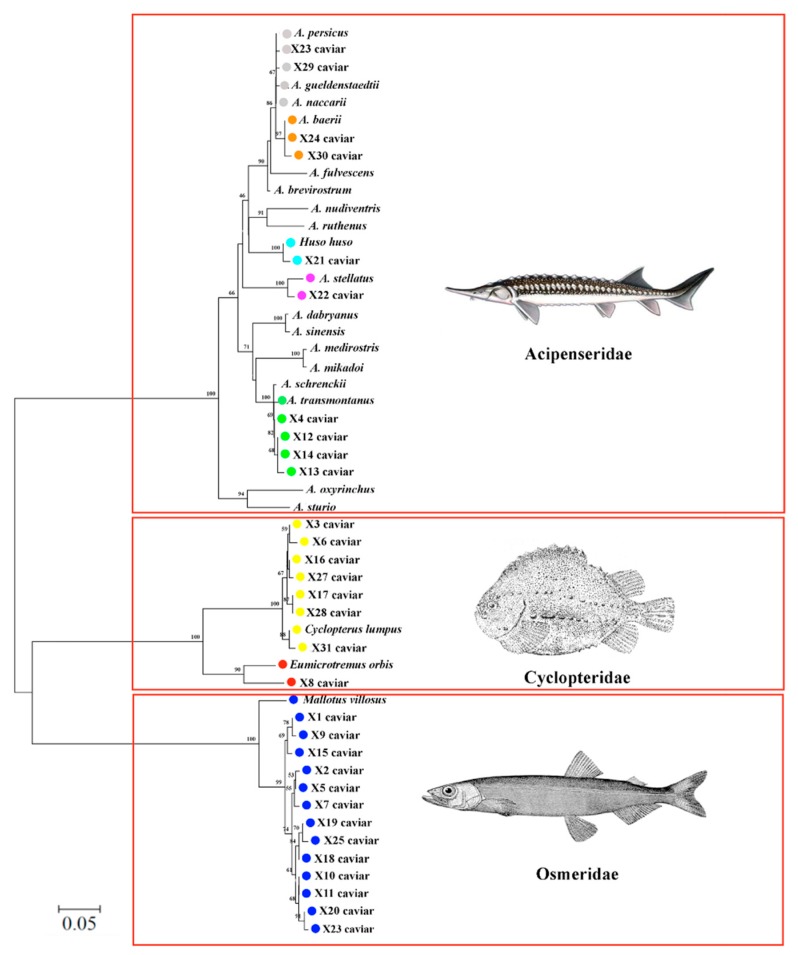
COI-neighbor-joining (CO-NJ) dendrogram showing the relationship of unknown sample sequences (X) to validated reference barcode sequences. The numbers above the nodes represent bootstrap analysis after 1000 replicates.

The COI barcode sequences of each species were the same length, ranging between species from 646 to 664 bp, without stop codons, and were functional mitochondrial sequences. Therefore, NUMT, (nuclear DNA sequences originating from mitochondrial DNA) were not sequenced (vertebrate NUMTs are generally smaller than 600 bp) [34]. The NJ tree (Figure 1) built using the sequences of the twenty species downloaded from GenBank (Table 1) and the sequences obtained from the commercial samples, identified by the BLAST search (Table 2), confirmed that the eight caviar species belong to three families: Acipenseridae (*H. huso*, *A. transmontanus*, *A. gueldenstaedtii*, *A. stellatus* and *A. baerii*), Cyclopteridae (*Cyclopterus lumpus* and *Eumicrotremus orbis*) and Osmeridae (*Mallotus villosus*). The nodes connecting the sequences belonging to the same species in the tree were well supported by high bootstrap values (>70%).

### 2.2. COIBar-RFLP

**Figure 2:**
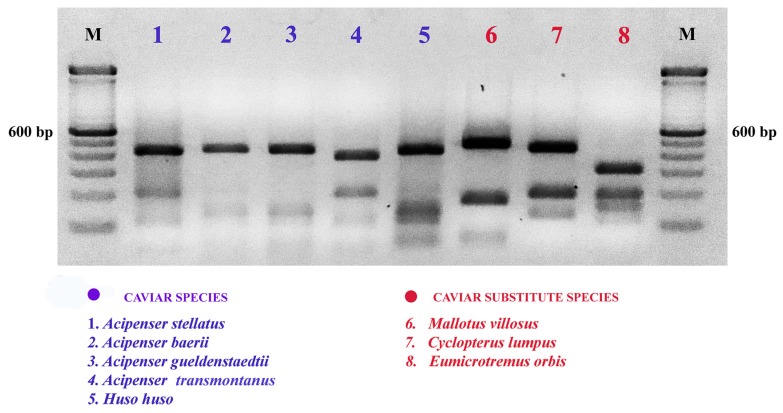
Example of COIBar-RFLP identification on a 3% agarose gel of caviar and substitute caviar species with restriction by *MboI* of the cytochrome c oxidase I gene. M = molecular weight marker (Trackit ^TM^ 100 bp DNA ladder, Invitrogen). All digestion experiments were conducted in triplicate.

PCR amplification of the mitochondrial COI gene of the examined species generated a fragment of variable size ranging from 700 to 760 bp. Unique restriction patterns of *Mbo*I for each species were found to satisfactorily differentiate between all eight tested species. The preliminary screening of suitable restriction enzymes generated using the EMBOSS (European Molecular Biology Open Software Suite) remap, showed that only *Mbo*I was able to simultaneously distinguish the different validated species of fish examined in this study. As illustrated in Figure 2, PCR product digestion resulted in specific profiles for the eight species: *E. orbis* has three *Mbo*I restriction sites and four fragments of about 300, 200, 150 and 100 bp. Five species have two restriction sites producing 410, 200 and 120 bp fragments in *A. stellatus*; 420, 140 and 100 bp in *A. gueldenstaedtii*; 380, 210 and 100 bp in *A. transmontanus;* 430, 150 and 110 bp in *H. huso* and finally 480, 220 and 130 bp in *C. lumpus*. Two species have only one *Mbo*I restriction site yielding 420 and 130 bp in *A. baerii* and 510 and 170 bp in *M. villosus*.

## 3. Discussion

The results obtained in this study provide insight into the identification of species in commercial caviar products using COI DNA barcoding. COIBar-RFLP, a method coupling the consolidated COI barcode and the random fragment length polymorphism techniques, was shown to effectively discriminate species by using the specific profiles of the *Mbo*I restriction enzyme. The COI gene sequences allowed the identification of four species of *Acipenser*, *H. huso* and three caviar substitute species, all forming well-supported clusters in the NJ tree. In this regard, our data showed the inclusion of *H. huso* within the genus *Acipenser* as already suggested by Birstein et al. [35] based on Cytb sequences. The authentication of caviar species is both a concern and a challenge to counter the illegal trade of caviar and sturgeon products as a high proportion of Acipenseriformes species are classified by the IUCN as “Critically Endangered” as a result of sturgeon habitat deterioration and overexploitation for caviar production [36]. For these reasons, resolution 12.7 (Rev. CoP17) of the Conference of the parties to CITES on “Conservation on and trade in sturgeons and paddlefish” established rules for controlling the trade, and recommended that the range of state-level export quotas for caviar and meat of Acipenseriformes species be followed. Furthermore, guidelines for a universal labeling system for the trade in and identification of caviar have also been established. On the other hand, our investigation of other brands highlighted that 14% of caviar products examined lacked a label declaring the name of the species from which the eggs originated. Worthy of note, using COIBar-RFLP, we succeeded in identifying the species *M. villosus* in almost all products uncorrectly labeled and one product resulted even contained the species *E. orbis*, belonging to Cyclopteridae, which is not on the list of fish species known to be used for caviar production [3]. One could speculate that a species commonly used as a caviar substitute, the lumpfish, *Cyclopterus lumpus*, another Cyclopteridae, was misidentified leading to unintentional fraud. However, this would not explain our case because the product was labeled as “caviar” and not as “lumpfish caviar”. Accurate species identification is very important for regulating trade of high value animal products such as sturgeon caviar. The most well-known and highly-prized caviars are Beluga from the Beluga sturgeon (*H. huso*), Osetra from the Russian sturgeon (*A. gueldenstaedtii*), and Sevruga from the Starry sturgeon (*A. stellatus*) [4]. However, most caviar on the market today is from the most common species, such as the White sturgeon (*A. transmontanus*) or the more highly-prized Siberian sturgeon (*A. baerii*). All of these species were correctly declared on the label of the caviar products examined here, probably because they derive from traceable aquaculture as stated on the website of the seller. In this regard, although our data indicate that the true caviar-based products were correctly labeled, poaching and illegal trade of sturgeon species have been recognized globally [37,38] in spite of trade limitations imposed by CITES. In our investigation, the most common species detected in caviar products sold in supermarkets was the capelin *M. villosus*, identified both in correctly-labeled products (declaring the words “caviar substitute” as required by the Italian law, art. 9 Reg. 1169/11) and mislabeled products (labeled as caviar only). The production of caviar substitutes and imitations was estimated to be about 50,000 t year^−1^ in 2011 due to the high demand of low price caviar by new consumers [3,39]. The consumption of products from caviar substitutes has grown considerably in recent years among large retailers and the capelin roe are the least expensive among the substitutes of true caviar, as their price is 1/3 lower than lumpfish caviar. Thus, a product not compliant with labeling guidelines would be easily considered as commercial fraud.

In this context, the identification of fish species using a molecular approach is the only way to determine if commercial fraud has occurred. Among the various molecular identification methodologies, PCR-RFLP is one of the most commonly used for fish species discrimination [40]. However, it should be noted that this method is very useful only when the commercial products contain single species. In the case of samples with mixed species, other techniques must be performed for taxonomic identification, such as next generation sequencing or species-specific multiplex PCR. Cytb gene sequencing and RFLP has been recommended by Ludwig [33,37,41] to discriminate caviar species, but the authors noted the limitations of this molecular marker for the differentiation of closely related species such as *A. gueldenstaedtii, A. baerii, A. persicus* and *A. naccarii* (the Russian complex species). More recently, PCR-RFLP of Cytb was also used by Trocchia et al. [42] to discriminate *A. baeri* from caviar substitute species such as *C. lumpus, M.villosus* and *Trisopterus minutus minutus*. In this case, Cytb RFLP produced a restriction pattern useful to differentiate the four species, but the discrimination within the genus *Acipenser* was not explored. Differently from PCR-RFLP of Cytb used by other researchers, our results based on COIBar-RFLP allowed us to simultaneously discriminate not only the four species of *Acipenser*, including *A. baerii* and *A. gueldenstaedtii* belonging to the Russian complex species and *H. huso*, but also the three caviar substitute species.

Recently, the COI barcode region has been coupled with high resolution melting analysis (Bar-HRM) for fish species discrimination [43]. However, this methodology requires experience for mini-barcode primer design and for testing their efficacy and specificity. Conversely, COIBar-RFLP does not require a high level of expertise in molecular genetics, has a very good performance in terms of ease and rapidity of execution (results of screening can be obtained in 7 h) and encourages further validation and application. We could explore building a database of restriction enzyme profiles to be used to validate this method, first identifying the species through the RFLP banding pattern and then confirming the species authentication by DNA sequencing. After validation, COIBar-RFLP could be used in routine screening for seafood product species authentication.

## 4. Materials and Methods

### 4.1. Sampling

A total of 31 different commercial caviar products were bought from several Italian markets and online websites in 2018. The fish species from which the eggs purportedly came was stated on the label of 27 out of 31 commercial products; the remaining products were labeled only as caviar. For each commercial brand, up to three eggs, for a total of 90 samples, were randomly chosen and independently processed to investigate the presence of multiple species in the caviar products (Table 2). Each sample was preserved in 1.5 mL labeled tubes filled with 95% ethanol, stored at −20 °C and subsequently processed in the laboratory.

### 4.2. COI Barcode Amplification, Sequencing and Data Analysis

Total genomic DNA was extracted from fish eggs using the DNeasy tissue kit (Qiagen, Hilden, Germany) following the manufacturer’s instructions with some modifications.

Cytochrome oxidase I amplifications were carried out in 20 µL volumes using the M13 tailed primers VF2_t1 and FishR2_t1 described in Ivanova et al. 9 [44] and the PCR conditions reported by Pappalardo and Ferrito [29]. Double-stranded products were checked by agarose gel electrophoresis, visualized with SYBR^®^ Safe (Thermo Fisher, Waltham, MA USA) and displayed through “Safe Imager ^TM^ 2.0 Blue Light Transilluminator” (Thermo Fisher, Waltham, MA USA). All PCR products were then purified with the QIAquick PCR purification kit (Qiagen) and subsequently sequenced in the forward and reverse direction by Genechron (http://www.genechron.it/index.php/sanger-sequencing) using M13 forward and M13 reverse primers. Sequence chromatograms obtained were checked visually, assembled and subsequently queried against the publicly accessible databases Basic Local Alignment Search Tool (BLAST, NCBI, Bethesda, MD USA) and the Barcode of Life Database (BOLD, http://www.boldsystems.org), with the species level barcode records selected [45]. All sequences derived from this study were deposited in GenBank (http://www.ncbi.nlm.nih.gov/genba nk/) (Table 2). The sequences were aligned using the default settings in ClustalX software [46] and the alignment was manually revised in BioEdit (http://www.mbio.ncsu.edu/bioedit/bioedit.html). MEGA v 6.0 software (Biodesign Institute, Arizona, MA, USA) [47] was used to construct a COI neighbor-joining (NJ) dendrogram using K2P distance. To validate the COI sequences of the species, another twenty COI sequences were downloaded from GenBank and BOLD SYSTEMS (http://www.boldsystems.org) and used to build the NJ tree (Table 1). Bootstrap values were calculated with 1000 replicates [48]. Ambiguous sequences were trimmed after alignment.

### 4.3. COIBar-RFLP

The restriction enzymes and their restriction sites were preliminarily detected using the “Remap” software (http://emboss.bioinformatics.nl) to scan several validated DNA barcoding sequences (650 bp) of reference caviar species downloaded from GenBank. The selected enzymes for carrying out the COI-RFLP analysis were: *Hpa*II (C*CGG), *Hinf*I (G*ANTC), *Mbo*I (*GATC) and *Rsa*I (GT*AC) (New England Biolabs, Inc., Ipswich, MA, USA). Then, the COI barcode PCR products of samples obtained from *Acipenser baerii* Brandt, 1869, *A. gueldenstaedtii* Brandt, 1833, *A. stellatus* Pallas, 1771, *A. transmontanus* Richardson, 1837, *Huso huso* L., 1758, *Mallotus villosus*, (Müller 1776), *Cyclopterus lumpus* Linnaeus, 1758**, and *Eumicrotremus orbis* (Günther, 1861) were digested with the selected restriction enzymes. A total volume of 15 μL containing 1 μL of enzyme buffer, 13 μL of unpurified PCR product and 1 μL of each endonuclease (10 U each) was used to carry out the digestion reaction. The reaction mixture was incubated in a water bath for 1 h at 37 °C. The digested PCR products were then separated on a 2.5% agarose gel and their sizes were determined by comparison with a Trackit ^TM^ 100 bp DNA ladder (Invitrogen). The pattern of bands obtained from the validated samples was used to identify the unknown commercial samples (figure not shown).

## 5. Conclusions

DNA-based techniques and a wide range of developed methodologies have become the most accurate molecular tools for fish species identification. However, to select a method to use in regular screening programs, several factors should be considered including complexity, cost and application range [49]. COIBar-RFLP has been used to discriminate the species of a wide number of taxonomic groups including fish at various stages of their life cycle (eggs, larval and adult) and as whole samples or processed samples, proving to be a reliable technique to fight commercial fraud based on unintentional or deliberate species substitutions in seafood products [29,30,31,32]. In addition, this method could also be successfully applied to fight illegal, unreported and unregulated fishing affecting critically-endangered species such as sturgeons.

## Figures and Tables

**Table 1:** GenBank accession numbers of the reference caviar and substitute fish species COI sequences.

Species	Common Name	GenBank Accession Number	References
*Acipenser baerii*	Siberian sturgeon	KP833625	Unpublished
*Acipenser brevirostrum*	Shortnose sturgeon	EU523870	Hubert et al. 2008
*Acipenser dabryanus*	Yangtze sturgeon	KP218558	Li et al. 2015
*Acipenser fulvescens*	Lake sturgeon	EU524392	Hubert et al. 2008
*Acipenser gueldenstaedtii*	Russian sturgeon	JQ623904	Unpublished
*Acipenser medirostris*	Green sturgeon	EU523879	Hubert et al. 2008
*Acipenser mikadoi*	Sakhalin sturgeon	NC031188	Unpublished
*Acipenser naccarii*	Adriatic sturgeon	KJ552424	Geiger et al. 2014
*Acipenser nudiventris*	Ship sturgeon	JQ 623905	Unpublished
*Acipenser oxyrinchus*	Atlantic sturgeon	KX145066	Unpublished
*Acipenser persicus*	Persian sturgeon	FJ809724	Unpublished
*Acipenser ruthens*	Sterlet	HQ960576	Unpublished
*Acipenser schrenckii*	Amur sturgeon	KP218530	Li et al. 2015
*Acipenser sinensis*	Chinese sturgeon	KP218545	Li et al. 2015
*Acipenser stellatus*	Stellate sturgeon	KC500131	Keskin & Atar 2013
*Acipenser sturio*	Common sturgeon	KJ552406	Geiger et al. 2014
*Acipenser transmontanus*	White sturgeon	EU523889	Hubert et al. 2008
*Huso huso*	Beluga sturgeon	EHSI214-06 *	Unpublished
*Mallotus villosus*	Capelin	HQ712650	Macklenburg et al. 2011
*Cyclopterus lumpus*	Lumpfish	MG421955	Unpublished
*Eumicrotremus orbis*	Pacific spiny lumpsucker)	JQ354090	Unpublished

* BOLD SYSTEM sequence ID.

**Table 2:** Samples of caviar and caviar substitute included in this work. In bold mislabeled seafood products.

Code	Processed Fish Product	n° Sample	Declared Species	GenBank Access N°	Species Matched by BLAST	Matched Accession from BLAST	% Identity
X1	capelin	3	*Mallotus villosus*	MK903699	*Mallotus villosus*	FJ205579	99.70
X2	capelin	3	*Mallotus villosus*	MK903700	*Mallotus villosus*	FJ205579	99.24
X3	lumpfish	3	*Cyclopterus lumpus*	MK903701	*Cyclopterus lumpus*	KJ204826	99.85
X4	white caviar	3	*Acipenser transmontanus*	MK903717	*Acipenser transmontanus*	KX145032	99.85
X5	black capelin	3	*Mallotus villosus*	MK903702	*Mallotus villosus*	FJ205579	99.39
X6	lumpfish	3	*Cyclopterus lumpus*	MK903703	*Cyclopterus lumpus*	KJ204826	99.54
X7	capelin	3	*Mallotus villosus*	MK903704	*Mallotus villosus*	FJ205579	99.09
X8	caviar	3	**not specified**	MK903718	*Eumicrotremus orbis*	JQ354090	96.91
X9	caviar	3	**not specified**	MK903705	*Mallotus villosus*	FJ205579	99.55
X10	red capelin	3	*Mallotus villosus*	MK903706	*Mallotus villosus*	FJ205579	99.09
X11	black capelin	3	*Mallotus villosus*	MK903706	*Mallotus villosus*	FJ205579	99.09
X12	white caviar	3	*Acipenser transmontanus*	MK903719	*Acipenser transmontanus*	KX145032	99.85
X13	white caviar	3	*Acipenser transmontanus*	MK903720	*Acipenser transmontanus*	KX145032	99.54
X14	white caviar	3	*Acipenser transmontanus*	MK903719	*Acipenser transmontanus*	KX145032	99.85
X15	capelin	3	*Mallotus villosus*	MK903707	*Mallotus villosus*	FJ205579	98.94
X16	lumpfish	3	*Cyclopterus lumpus*	MK903708	*Cyclopterus lumpus*	KJ204826	99.69
X17	lumpfish	3	*Cyclopterus lumpus*	MK903709	*Cyclopterus lumpus*	KJ204826	99.38
X18	red capelin	3	*Mallotus villosus*	MK903710	*Mallotus villosus*	FJ205581	98.94
X19	caviar	3	**not specified**	MK903711	*Mallotus villosus*	FJ205581	98.79
X20	capelin	3	*Mallotus villosus*	MK903712	*Mallotus villosus*	FJ205579	98.79
X21	beluga caviar	2	*Huso huso*	MK903721	*Huso huso*	AY442351	99.69
X22	sevruga caviar	2	*Acipenser stellatus*	MK903722	*Acipenser stellatus*	HQ960585	99.08
X23	oscietra caviar	2	*Acipenser gueldenstaedtii*	MK903723	*Acipenser gueldenstaedtii*	KC500088	99.85
X24	baeri caviar	3	*Acipenser baerii*	MK903724	*Acipenser baerii*	KM286420	99.85
X25	caviar	3	**not specified**	MK903713	*Mallotus villosus*	FJ205581	98.49
X26	red capelin	3	*Mallotus villosus*	MK903714	*Mallotus villosus*	FJ205579	98.64
X27	lumpfish	3	*Cyclopterus lumpus*	MK903715	*Cyclopterus lumpus*	KJ204826	99.54
X28	lumpfish	3	*Cyclopterus lumpus*	MK903709	*Cyclopterus lumpus*	KJ204826	99.38
X29	oscietra caviar	3	*Acipenser gueldenstaedtii*	MK903725	*Acipenser gueldenstaedtii*	KC500088	99.85
X30	baeri caviar	3	*Acipenser baerii*	MK903726	*Acipenser baerii*	KM286420	99.69
X31	lumpfish	3	*Cyclopterus lumpus*	MK903716	*Cyclopterus lumpus*	KJ204826	99.38
